# A new species of *Tylototriton* (Amphibia, Salamandridae) from Phu Xai Lai Leng Mountain, Nghe An Province, Vietnam

**DOI:** 10.3897/zookeys.1276.173848

**Published:** 2026-04-08

**Authors:** An Vinh Ong, Tien Quang Phan, Chung Van Hoang, Mai Hong Thi Nguyen, Tao Thien Nguyen, Thomas Ziegler, Truong Quang Nguyen, Cuong The Pham

**Affiliations:** 1 Department of Zoology, Vinh University, 182 Le Duan Road, Nghe An, Vietnam Institute of Zoology, University of Cologne Cologne Germany; 2 Institute of Biology, Vietnam Academy of Science and Technology, 18 Hoang Quoc Viet Road, Hanoi 10072, Vietnam Institute of Biology, Vietnam Academy of Science and Technology Hanoi Vietnam; 3 Graduate University of Science and Technology, Vietnam Academy of Science and Technology, 18 Hoang Quoc Viet Road, Hanoi 10072, Vietnam Graduate University of Science and Technology, Vietnam Academy of Science and Technology Hanoi Vietnam; 4 Cologne Zoo, Riehler Str. 173, D-50735 Cologne, Germany Department of Zoology, Vinh University Nghe An Vietnam; 5 Institute of Zoology, University of Cologne, Zülpicher Str. 47b, D-50674 Cologne, Germany Cologne Zoo Cologne Germany

**Keywords:** Crocodile newts, morphology, ND2 gene, phylogenetic relationships, taxonomy, *Tylototriton vietnamirabilis* sp. nov.

## Abstract

A new species of crocodile newt, *Tylototriton vietnamirabilis***sp. nov**., is described from Phu Xai Lai Leng Mountain, Nghe An Province, in the border area between Vietnam and Laos, based on molecular divergence and morphological differences. *Tylototriton vietnamirabilis***sp. nov**. differs from other species in the subgenus *Tylototriton* by its body size, tail length, glandular ridge on the midline of crown of head, parotoid shape, appearance of vertebral ridge, number of dorsolateral glandular warts, the presence of a gular fold, coloration of head and body, and the presence of lateral grooves on tail. In terms of genetic distance, the new species differs from other congeners for which comparable sequences are available by at least 5.33–5.35% (*T. panwaensis*) and 5.35–5.37% (*T. anguliceps*), based on the mitochondrial NADH dehydrogenase subunit 2 (ND2) gene. Our new finding brings the total number of known species in the genus *Tylototriton* from Vietnam to 10. Because the new species is currently known to be restricted to evergreen montane forests on Phu Xai Lai Leng Mountain, we recommend to be classified as Endangered (EN) on the IUCN Red List.

## Introduction

The members of *Tylototriton* Anderson, 1871, commonly known as crocodile newts, inhabit montane forest areas and are distributed across Asia, from north-central India, eastern Nepal, central to southern China (including Hainan Island), Bhutan, Myanmar, southwards through Laos, Vietnam and Thailand (Frost 2025). The genus *Tylototriton* currently comprises 43 species, 17 of which have been described in the last five years (Frost 2025). The genus was divided into three subgenera: *Tylototriton*, *Yaotriton*, and *Liangshantriton* (Bernardes et al. 2020; Pomchote et al. 2021, 2024), and includes several unnamed taxa that contain cryptic species that are morphologically difficult to distinguish (Hernandez 2016; Pomchote et al. 2021; Poyarkov et al. 2021; Phung et al. 2023). Most of the recent discoveries derived from the splitting of widely distributed taxa through the efforts of integrative taxonomy, namely, the combination of morphological and phylogenetic analyses.

In Vietnam, nine species of the genus are recognized, and five of them have been described in the past five years, viz. *Tylototriton pasmansi* Bernardes, Le, Nguyen, Pham, Pham, Nguyen & Ziegler, 2020; *T. sparreboomi* Bernardes, Le, Nguyen, Pham, Pham, Nguyen & Ziegler 2020; *T. thaiorum* Poyarkov, Nguyen & Arkhipov, 2021; *T. ngoclinhensis* Phung, Pham, Nguyen, Ninh, Nguyen, Bernardes, Le, Ziegler & Nguyen, 2023; and *T. koliaensis* Poyarkov, Nguyen, Le, Le, Arkhipov, Gorin, Hernandez & Dufresnes, 2024 (Bernardes et al. 2020; Poyarkov et al. 2021, 2024; Phung et al. 2023). The sixth species, *Tylototriton obsti* Bernardes, Le, Nguyen, Pham, Pham, Nguyen & Ziegler 2020, was described as a subspecies of *Tylototriton pasmansi* but it was recently recognized as a full species (Raffaëlli 2022; Decemson et al. 2023; Poyarkov et al. 2024).

Recent fieldwork on Phu Xai Lai Leng Mountain, Nghe An Province, north-central Vietnam has revealed a previously overlooked population of *Tylototriton*. This population shows notable genetic divergence and morphological differences from other known species in the genus. Thus, we herein describe the newly discovered crocodile newt population from Nghe An as a new species.

## Materials and methods

### Sampling

**Figure 1.**
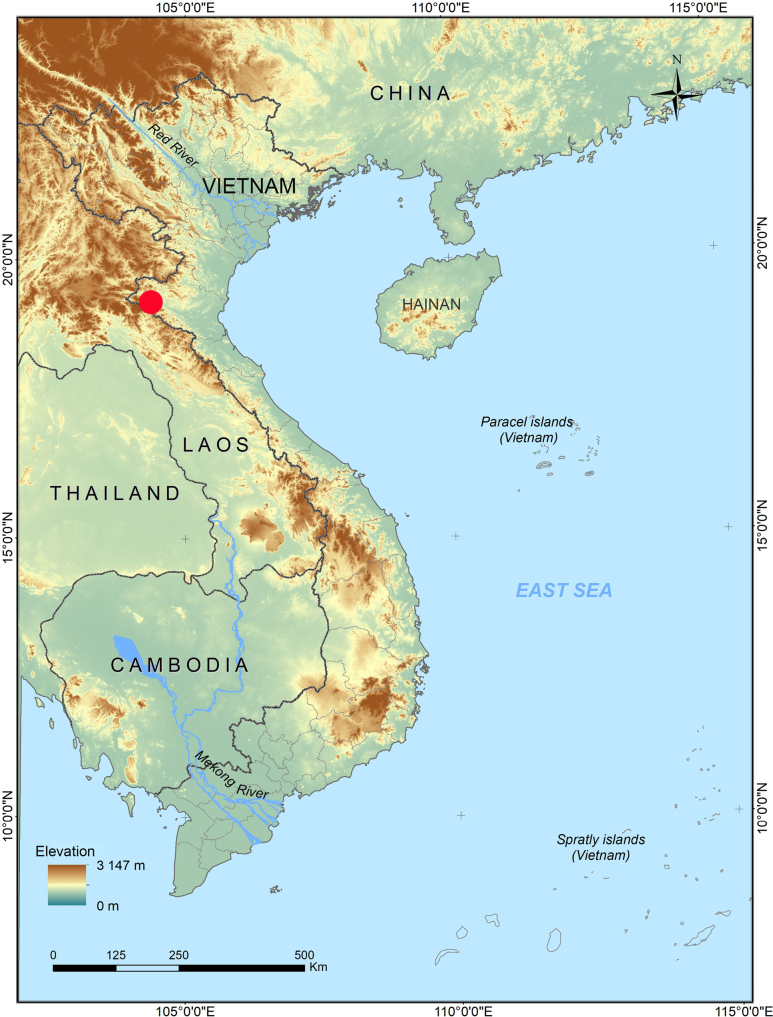
Type locality of *Tylototriton vietnamirabilis* sp. nov. in Phu Xai Lai Leng Mountain (red dot), Nghe An Province, Vietnam.

A field survey was conducted in July 2025 in the evergreen forest of Phu Xai Lai Leng Mountain, Nghe An Province, in the border area between Vietnam and Laos (Fig. 1). Phu Xai Lai Leng is known as one of the highest mountains in the Truong Son Range, rising to an elevation of 2720 m above sea level (Sterling et al. 2006). Straddling the international border between Laos and Vietnam, it ranks among Southeast Asia’s most prominent peaks (Sterling et al. 2006). After being photographed in life, crocodile newts were anaesthetized and euthanized in a closed vessel with a piece of cotton wool containing ethyl acetate (Simmons 2002), fixed in 80% ethanol for 5 h, and then transferred to 70% ethanol for permanent storage. Liver tissue samples were taken and preserved separately in 70% ethanol prior to fixation. Voucher specimens referred to in this paper were deposited in the collections of the Institute of Biology (IB), Hanoi, Vietnam.

### Molecular data and phylogenetic analyses

Tissue samples were extracted using the PureLink™ RNA Micro Scale Kit (Thermo Fisher Scientific), following the manufacturer’s instructions. Genomic DNA was amplified using an Applied Biosystems PCR machine. The PCR total volume was 25 μl, consisting of 12 μl of mastermix, 6 μl of water, 1 μl of each primer at a concentration of 10 pmol/μl, and 5 μl of DNA. Primers used in the PCR and sequencing were as follows: the primer pair Met-LND2 (5’-CAATGTTGGTTAAAATCCTTCC-3’) and TrpeHND2 (5’-AGGCTTTGAAGGCCTTTGGTC-3’) (Stuart et al. 2006), which was used to amplify a fragment of the NADH dehydrogenase subunit 2 (ND2) gene. PCR products were sent to Apical Scientific (Malaysia) for sequencing (https://apicalscientific.com). The obtained sequences were deposited in GenBank under accession numbers PX668411–PX668415 (Suppl. material 1).

In addition to sequences generated for five samples of the new population from Nghe An Province and two samples of *Tylototriton ngoclinhensis*, we used ND2 gene data from 17 samples of 15 species of the subgenus *Tylototriton*, a sample of *T. panhai* of the subgenus *Yaotriton*, a sample of *T. taliangensis* of the subgenus *Liangshantriton* and *Echinotriton andersoni*, which were included as the outgroups available from GenBank (Pomchote et al. 2024) for phylogenetic analyses. Localities and accession numbers of all sequences used in the study can be found in Suppl. material 1.

Chromas Pro software (Technelysium Pty Ltd, Tewantin, Australia) was used to edit the sequences, which were then aligned using ClustalW (Thompson et al. 1997) as embedded in MEGA11 (Tamura et al. 2021) with default parameters and subsequently optimized manually in BioEdit v. 7.0.5.2 (Hall 1999). Pairwise comparisons of uncorrected sequence divergence (p-distance) were calculated using MEGA11 (Tamura et al. 2021). Variance was estimated using the bootstrap method with 1000 replicates using nucleotide substitution while gap/missing data were treated via complete deletion.

Prior to Bayesian analyses, the optimal nucleotide substitution models for ND2 partitions were selected using Kakusan 4 (Tanabe 2011), based on the Akaike information criterion (AIC). Bayesian inference (BI) was estimated using MrBayes v .3.2 (Ronquist et al. 2012) under the GTR+G model. Two independent runs of four Markov Chains, three heated and one cold, were performed for 10,000,000 generations. Trees were sampled every 100 generations, and a consensus topology was calculated using 70,000 trees after discarding the first 30.000 trees (burnin = 3,000,000). We checked parameter estimates and convergence using Tracer v. 1.7.1 (Rambaut et al. 2018). For maximum likelihood (ML) analysis, IQ-TREE v. 1.6.12 (Nguyen et al. 2015) with the GTR+F+I+G4 model was used with 10,000 ultrafast bootstrap replications (UFB) (Hoang et al. 2018). We considered Bayesian posterior probability (BPP) and ultrafast bootstrap (UFB) support values of greater than or equal to 0.95 for BPP and 95% for UFB as strong support for a clade (Ronquist et al. 2012; Hoang et al. 2018).

### Morphological comparisons

A total of 28 morphological characteristics were measured on preserved specimens using a digital caliper to the nearest 0.1 mm, following Bernardes et al. (2020) and Phung et al. (2023), as follows:

**SVL** snout-vent length (measured from tip of snout to anterior tip of vent),

**HL** head length (measured from tip of snout to the gular fold),

**HW** head width (measured across the widest distance across the parotoid glands),

**SL** snout length (measured from tip of snout to the anterior corner of eye),

**PL** parotoid length,

**PW** parotoid width,

**PH** maximum parotoid height,

**EL** eye length (distance between anterior and posterior angles),

**EN** eye-narial distance (minimum distance between eyelid and nostril),

**IN** inter-narial distance (minimum distance between the external nares),

**IE** inter-eye distance (minimum distance between eyelids),

**LJL** lower jaw length (from tip of lower jaw to jaw angle),

**UEW** upper eyelid width (greatest width of upper eyelid),

**HUM** humerus length (distance from axilla to tip of elbow),

**RAD** radius length (distance from elbow to longest finger),

**FEM** femur length (distance from groin to knee),

**TIB** tibia length (distance from knee to tip of longest toe),

**FORE** total forelimb length,

**HIND** total hindlimb length,

**TL** tail length (measured from anterior of vent to tail tip),

**TH** tail height (measured at the medial section of tail),

**CIL** cloaca length (length of cloaca muscle),

**CIW** cloaca width (width of cloaca muscle),

**WVr** width of vertebral cord measured at the height of the 5^th^ nodule,

**L5W** length of the 5^th^ anterior dorsal nodule,

**AG** distance between axilla and groin,

**TKL** trunk length from wrinkle of throat (gular fold) to anterior tip of vent,

**TOL** total length (measured from tip of snout to tip of tail).

Morphological comparisons between the new taxon and its congeners were based on the specimen examinations and the following literature: Nussbaum et al. (1995), Zhao et al. (2012), Nishikawa et al. (2013, 2014, 2015), Khatiwada et al. (2015), Le et al. (2015), Phimmachak et al. (2015), Fei and Ye (2016), Hernandez (2016), Grismer et al. (2018, 2019), Zaw et al. (2019), and Pomchote et al. (2020, 2021, 2024).

To remove the effects of allometry in the following comparisons, morphometric data of the measurements were normalized to adjust raw morphometrics through the allom function in the R package GroupStruct (available at https://github.com/chankinonn/GroupStruct). Accordingly, the allometric formula is X_adj_ = log_10_(X) – ß[log_10_(SVL) – log_10_(SVL_mean_)], where X_adj_ = adjusted value for character X; X = measured value for character X; β = unstandardized regression coefficient for log(X) against log (SVL) and SVL_mean_ = grand mean of SVL (Thorpe 1975, 1983; Turan 1999; Lleonart et al. 2000; Chan and Grismer 2022). Principal component analysis (PCA) of the adjusted morphometric characters (i.e., HL, HW, IN, FORE, HIND, TL, CIL, AG, and TKL) was implemented by the prcomp () command, using the R package FactorMineR (Le et al. 2008) and visualized using the Factoextra and ggplot2 packages (Wickham 2016; Kassambara and Mundt 2020). One-way analyses of variance (ANOVAs) were performed to examine the interspecific differences in the raw snout-vent length (SVL), adjusted variables, and the first two principal components (PC1 and PC2) among species. ANOVAs with a p-value less than 0.05, indicating significant differences, were subjected to Tukey’s honest significant difference (HSD) test for comparisons among species pairs.

## Results

### Phylogenetic analyses

The combined matrix of ND2 contained 1001 aligned characters, of which 645 were conserved and 356 sites exhibited variation, of which 213 were found to be potentially parsimony informative. The overall transition/transversion bias is *R* = 3.739. The nucleotide frequencies were 37.65% (A), 23.64% (T/U), 28.26% (C), and 10.45% (G).

**Figure 2.**
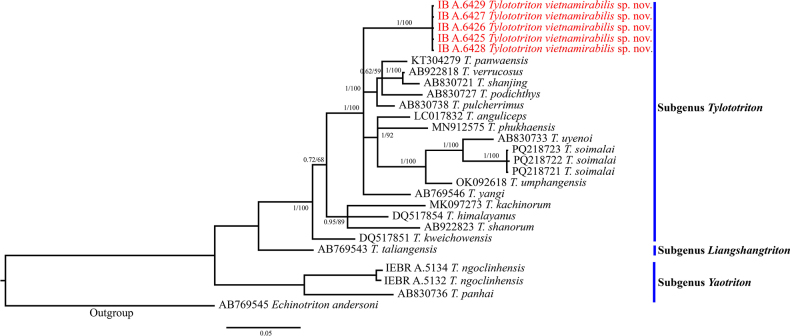
BI tree from a 1001 bp sequence of the mitochondrial ND2 gene for *Tylototriton* and outgroup species; ML ultrafast bootstrap replications (UFB) and Bayesian posterior probabilities (BPP) are shown in this order near the node. For GenBank accession numbers, refer to Suppl. material 1.

Phylogenetic analyses employing ML and BI methods were nearly identical, with most well-supported nodes on the ML tree also well-supported on the BI tree; only the BI tree is presented in Fig. 2. The unnamed taxon from Nghe An Province is strongly supported as a member of the subgenus *Tylototriton* and as a sister clade to *T. panwaensis*, *T. verrucosus*, *T. shanjing* Nussbaum, Brodie & Yang, 1995, *T. pulcherrimus*, *T. podichthys* Phimmachak, Aowphol & Stuart, 2015, *T. anguliceps*, *T. phukhaensis* Pomchote, Khonsue, Thammachoti, Hernandez, Peerachidacho, Suwannapoom, Onishi & Nishikawa, 2020, *T. uyenoi*, *T. soimalai*, *T. umphangensis* Pomchote, Peerachidacho, Hernandez, Sapewisut, Khonsue, Thammachoti & Nishikawa, 2021 and *T. yangi* Hou, Zhang, Zhou, Li & Lu, 2012 (BPP = 1, UFB = 100) (Fig. 2).

In terms of pairwise genetic distance based on ND2 data, interspecific uncorrected p-distance of the subgenus *Tylototriton* species ranged from 2.03% (between *T. verrucosus* Anderson, 1871 and *T. pulcherrimus* Hou, Zhang, Li & Lu, 2012) to 10.72% (between *T. shanorum* Nishikawa, Matsui & Rao, 2014 and *T. soimalai* Pomchote, Peerachidacho, Khonsue, Sapewisut, Hernandez, Phalaraksh, Siriput & Nishikawa, 2024) (Table 1). The genetic divergence between the new population from Nghe An Province, Vietnam, and its congeners ranged from 5.33–5.35% (*T. panwaensis*) to 10.43–10.53% (*T. kachinorum*) (Table 1).

**Table 1.** Mean uncorrected (p) distance (%) among 1001 bp fragments of ND2 of the subgenus *Tylototriton* and related taxa.

	Species	1	2	3	4	5	7	8	9	9	10	11	12	13	14	15	16	17	18	19
1	*Tylototriton vietnamirabilis* sp. nov.	**0**																		
2	*T. panwaensis*	5.33–5.35	**0**																	
3	*T. anguliceps*	5.35–5.37	4.69	**0**																
4	*T. phukhaensis*	6.68–6.71	6.01	4.58	**0**															
5	*T. verrucosus*	5.45–5.47	2.24	4.48	5.12	**0**														
6	*T. kweichowensis*	7.23–7.26	5.66	6.23	6.45	5.43	**0**													
7	*T. kachinorum*	10.43–10.53	6.79	8.33	9.35	7.5	6.74	**0**												
8	*T. himalayanus*	8.08–8.12	6.92	7.28	7.74	6.58	5.56	5.57	**0**											
9	*T. shanorum*	8.69–8.73	6.96	7.43	8.68	6.6	6.47	8.32	5.49	**0**										
10	*T. yangi*	6.41–6.43	4.14	4.35	6	3.93	6.31	8.8	7.01	7.39	**0**									
11	*T. podichthys*	6.45–6.47	3.51	5.13	6.25	3.51	5.8	8.6	7.17	8.03	5.23	**0**								
12	*T. pulcherrimus*	5.38–5.40	2.65	4.25	4.7	2.03	5.33	8.28	6.69	7.07	3.92	3.29	**0**							
13	*T. shanjing*	5.74–5.77	3.23	4.65	5.63	0.92	5.85	8.52	6.44	7.04	4.63	4.1	2.58	**0**						
14	*T. uyenoi*	9.71–9.78	8.15	7.81	7.84	7.61	8.77	13.11	9.76	10.4	8.28	9.25	7.26	8.06	**0**					
15	*T. umphangensis*	7.55–7.58	5.77	5.44	6.55	5.13	8.24	9.64	8.64	9.01	5.99	7.46	5.98	5.53	5.1	**0**				
16	*T. soimalai*	9.86–10.02	8.59	7.45	8.38	7.8	9.32	12.28	10.55	10.72	8.35	8.97	7.24	8.04	4.24	6.06	**0.00–0.10**			
17	*T. taliangensis*	9.72–9.76	7.86	8.81	9.04	7.41	6.48	7.86	7.87	8.72	8.19	8.12	7.41	7.52	10.76	9.86	10.51	**0**		
18	*T. ngoclinhensis*	13.08–13.51	12.68	13.33	13.31	12.14	10.75	13.65	11.91	12.33	12.4	11.98	11.92	12.35	15.31	14.81	10.59	14.65–15.31	**0**	
19	*T. panhai*	14.81–14.88	14.27	14.82	14.58	14.02	11.6	15.49	13.77	14.11	14.22	14.09	13.4	14.38	16.51	15.96	11.49	15.94–16.08	7.28–7.29	**0**

### Morphological analysis

**Figure 3.**
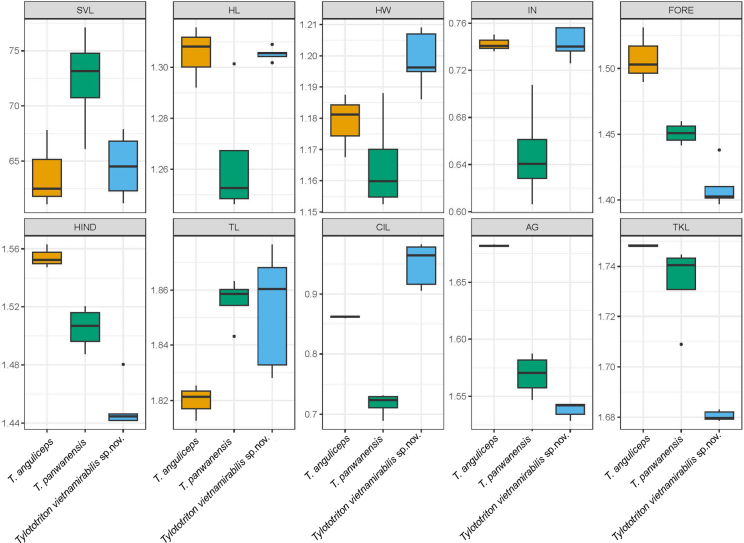
Boxplot comparisons of the snout-vent length (SVL) and size-adjusted morphometric characters among *Tylototriton vietnamirabilis* sp. nov. and *T. panwaensis* and *T. anguliceps*. Abbreviations are defined in the Materials and methods.

Based on the phylogenetic analyses, the new species was placed within the genus *Tylototriton*, but without a clear sister taxon, and data on previously known species were limited. Therefore, only two closely related taxa in the genus *Tylototriton* were used for comparison in this analysis, including *Tylototriton anguliceps* Le, Nguyen, Nishikawa, Nguyen, Pham, Matsui, Bernardes & Nguyen, 2015 (Le et al. 2015) and *Tylototriton panwaensis* Grismer, Wood, Quah, Thura, Espinoza & Murdoch, 2019 from Kachin State, Myanmar (Grismer et al. 2019). The ANOVA results showed significant differences in snout-vent length (SVL) and size-adjusted variables among the three species (P-values < 0.05). The post-hoc tests indicated that the new species significantly differed from *T. anguliceps* in FORE, HIND, TL, CIL, AG, and TKL, and from *T. panwaensis* in SVL, HW, IN, FORE, HIND, and CIL (P-values < 0.05) (Fig. 3).

**Figure 4.**
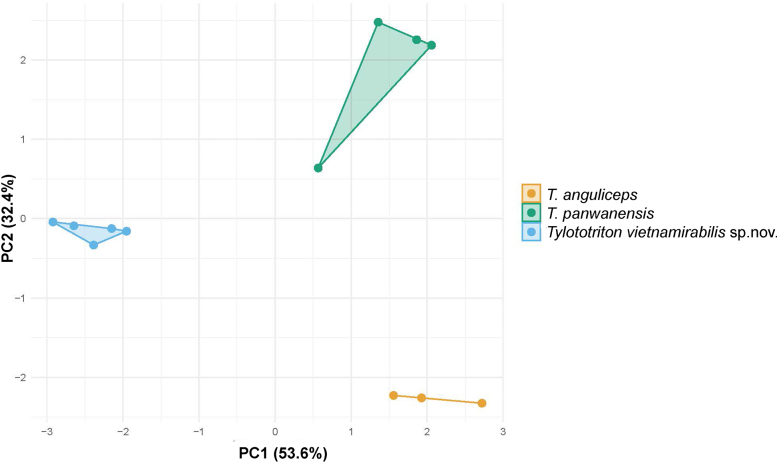
PCA of the size-adjusted morphometric characters among *Tylototriton vietnamirabilis* sp. nov. and *T. panwaensis* and *T. anguliceps*.

Only the first two principal components (PC1 and PC2) had eigenvalues greater than 1 and together accounted for 86.0% of the total variance (53.6% and 32.4%, respectively). The new species and two remaining species of *T. anguliceps* and *T. panwaensis* occupy separate locations in morphospace along the combined ordination of PC1 and PC2 (Fig. 4). ANOVAs and post-hoc tests demonstrated that the first two PCs’ values of *Tylototriton vietnamirabilis* sp. nov. differ significantly from those of *T. anguliceps* and *T. panwaensis* (P-values < 0.0001).

Therefore, we herein describe the population of *Tylototriton* from Nghe An Province as a new species.

### Taxonomic account

#### Tylototriton vietnamirabilis sp. nov.

Taxon classificationAnimaliaCaudataSalamandridae

DCBE9F38-90CD-5DD2-ACF7-1531BCC1852A

https://zoobank.org/BCFA5A10-32A2-4837-A818-037CEC8071F8

**Figure 5.**
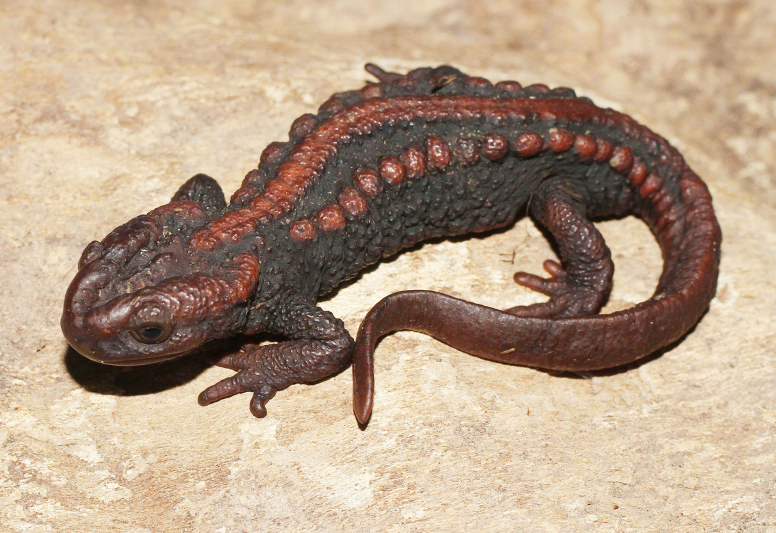
Dorsolateral view of the male holotype of *Tylototriton vietnamirabilis* sp. nov. (IB A.6425) in life. Photo: CTP.

**Figure 6.**
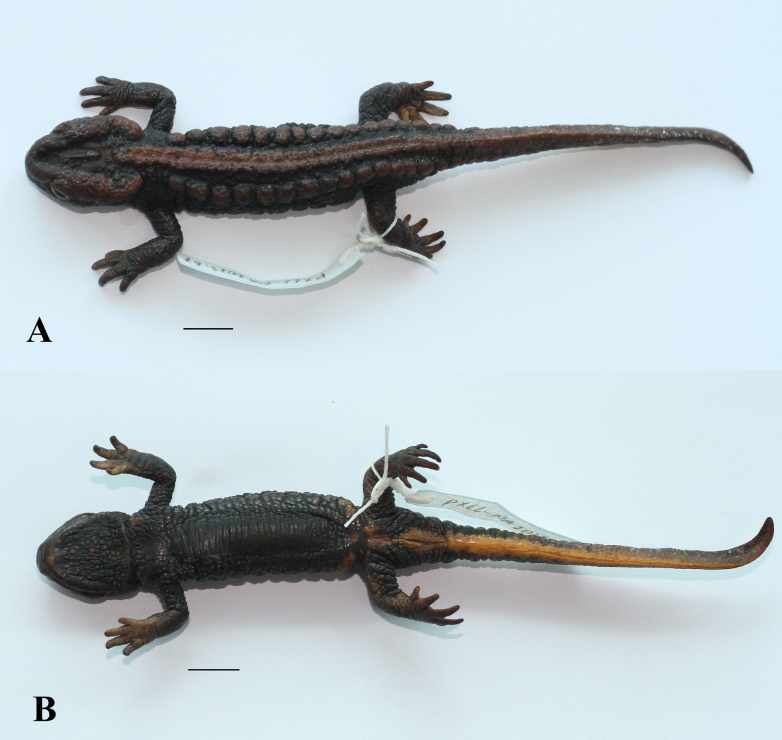
Holotype of *Tylototriton vietnamirabilis* sp. nov. (IB A.6425, male) in preservative. **A**. Dorsolateral view; **B**. Ventral view. Scale bars: 10 mm. Photos: CTP.

**Figure 7.**
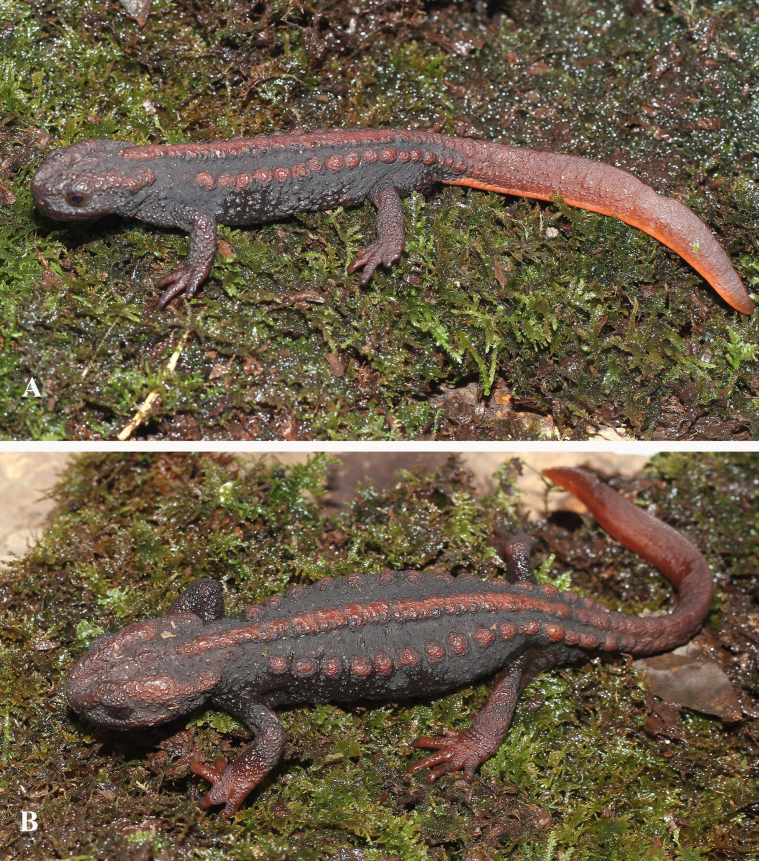
Paratypes of *Tylototriton vietnamirabilis* sp. nov. in life. **A**. Dorsolateral view (IB A.6427, male); **B**. Dorsolateral view (IB A.6429, female). Photos: CTP.

**Figure 8.**
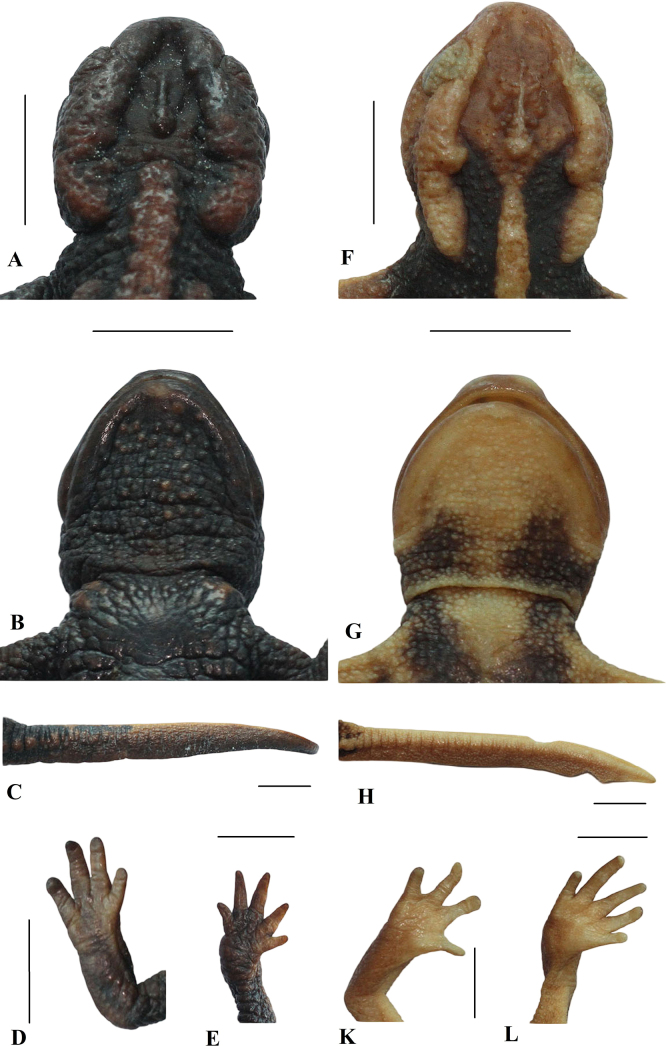
Holotype of *Tylototriton vietnamirabilis* sp. nov. (IB A.6425, male) in preservative. **A**. Dorsolateral view of head; **B**. Ventral view of head; **C**. Lateral view of tail; **D**. Ventral view of right hand; **E**. Ventral view of right foot. *Tylototriton anguliceps* (IB A.6434, male) in preservative. **F**. Dorsolateral view of head; **G**. Ventral view of head; **H**. Lateral view of tail; **K**. Ventral view of right hand; **L**. Ventral view of right foot. Scale bars: 10 mm. Photos: CTP.

Figs 5, 6, 7, 8, Table 2

##### Type material.

***Holotype***. • IB A.6425 (Field No. PXLL-NA 2025.67), adult male collected by A. V. Ong, T. Q. Phan, and N. H. Nguyen on 4 July 2025 in the evergreen forest of Phu Xai Lai Leng Mountain (19°23.483'N, 104°09.978'E, at an elevation of 1884 m a.s.l.), Nghe An Province, Vietnam. ***Paratypes***. • Four adult males: IB A.6426 (Field No. PXLL-NA 2025.68), IB A.6427 (Field No. PXLL-NA 2025.64), IB A.6428 (Field No. PXLL-NA 2025.65), IB A.6429 (Field No. PXLL-NA 2025.66); • one adult female: IB A.6430 (Field No. PXLL-NA 2025.69), the same collection data as the holotype.

**Table 2.** Morphometric measurements (mm) of the type series of *Tylototriton vietnamirabilis* sp. nov.

Voucher	IB A.6425	IB A.6426	IB A.6427	IB A.6428	IB A.6429	Min.–Max.	Mean ± SD	IB A.6430
Sex	M	M	M	M	M	(*N* = 5)	(*N* = 5)	F
Type status	H	P	P	P	P			P
SVL	66.8	67.9	62.3	61.2	64.5	61.2–67.9	64.54 ± 2.85	72.6
HL	21.3	21.4	19.3	18.8	20.2	18.8–21.4	20.20 ± 1.16	22.5
HW	16.5	16.8	14.8	14.3	16.1	14.3–16.8	15.70 ± 1.09	17.6
SL	6.4	6.5	6.4	5.9	6.2	5.9–6.5	6.28 ± 0.24	7.1
PW	5.4	5.5	4.8	4.5	4.8	4.5–5.5	5.00 ± 0.43	6.0
PL	14.1	13.8	13.6	13.2	14.4	13.2–14.4	13.82 ± 0.46	14.5
PH	5.1	5.2	4.5	4.6	4.8	4.5–5.2	4.84 ± 0.30	5.7
EL	4.8	4.6	4.1	4.0	4.5	4.0–4.8	4.40 ± 0.34	5.6
EN	4.2	4.1	3.6	3.5	4.2	3.5–4.2	3.92 ± 0.34	5.3
IN	5.8	5.9	5.4	4.9	5.7	4.9–5.9	5.54 ± 0.40	6.5
IE	4.3	4.1	3.4	3.5	3.8	3.8–4.3	3.82 ± 0.38	4.8
LJL	15.1	14.1	13.2	12.6	14.2	12.6–15.1	13.84 ± 0.97	15.9
UEW	2.6	2.5	2.2	2.3	2.4	2.2–2.6	2.40 ± 0.16	2.8
HUM	8.3	8.4	8.2	8.4	9.3	8.2–9.3	8.52 ± 0.44	10.2
RAD	17.6	17.9	16.8	15.5	18.1	15.5–18.1	17.18 ± 1.06	18.6
FEM	9.5	9.6	8.5	8.6	9.5	8.5–9.6	9.14 ± 0.54	10.5
TIB	19.2	19.8	18.4	17.5	20.7	17.5–20.7	19.12 ± 1.24	21.6
FORE	25.9	27.5	25.0	23.9	27.4	23.9–27.5	25.94 ± 1.55	28.8
HIND	28.7	29.4	26.9	26.1	30.1	26.1–30.1	28.24 ± 1.69	32.1
TL	74.7	71.1	71.6	64.3	75.2	64.3–75.2	71.38 ± 4.36	78.3
TH	8.8	9.1	7.2	7.4	8.5	7.2–9.1	8.20 ± 0.85	9.7
CIL	9.4	8.5	9.4	7.8	9.5	7.8–9.5	8.92 ± 0.75	5.8
CIW	4.4	4.5	4.2	4.3	5.4	4.2–5.4	4.56 ± 0.48	3.6
WVr	5.4	5.2	4.6	4.7	5.4	4.6–5.4	5.06 ± 0.38	5.5
L5W	4.6	5.1	3.9	3.8	4.3	3.8–5.1	4.34 ± 0.53	4.8
AG	34.8	36.5	33.8	32.7	34.9	32.7–36.5	34.54 ± 1.41	38.2
TKL	49.8	50.3	46.5	45.2	47.8	45.2–50.3	47.92 ± 2.16	51.7
TOL	141.5	139	133.9	125.5	139.7	125.5–141.5	135.90 ± 6.40	150.9

##### Etymology.

The name “*vietnamirabilis*” is a Latinized form of the modern word creation *Vietnamazing*. The new species is named to highlight the Vietnamazing conservation campaign 2024–2025 of the European Association of Zoos and Aquariums (EAZA). The Vietnamazing campaign aimed to raise public awareness of Vietnam’s unique biodiversity and its conservation, establish conservation projects with crocodile newts being one of the flagship groups of the campaign and raise funds for species conservation, following and higlighting IUCN's "One Plan Approach to Conservation". From 2026 onwards, after the completion of the EAZA campaign, Vietnamazing will continue as the Vietnamazing conservation network, a program under ZGAP (Zoological Society for the Conservation of Species and Populations). As common names, we suggest Vietnamazing Crocodile Newt (English), Cá cóc việt nam kỳ thú (Vietnamese).

##### Diagnosis.

The new species is assigned to the subgenus *Tylototriton* based on the results of the molecular phylogenetic analyses and the following morphological attributes: back with dorsal granules, head with dorsolateral bony ridges, knob-like warts or rib nodules present on dorsolateral body, and the absence of quadrate spine (Le et al. 2015; Fei and Ye 2016; Pomchote et al. 2021, 2024). The new species is diagnosed from its congeners by a combination of the following morphological characteristics: (1) size medium (SVL 61.2–67.9 mm, TL 64.3–75.2 mm in males, and SVL 71.6 mm, TL 78.3 mm in a single female); (2) tail longer than the snout-vent length; (3) head longer than wide; (4) glandular ridge on midline of crown distinct; (5) parotoids prominent and enlarged, projecting backwards; (6) vertebral ridge large, raised, and glandular in appearance; (7) 15 or 16 distinct dorsolateral glandular warts; (8) gular fold present; (9) dorsolateral bony ridges, parotoids, rib nodules, and vertebral ridge dull orange; and (10) tail with distinct lateral grooves.

##### Description of holotype.

Size medium (SVL 66.8 mm, TL 74.7 mm); head longer than wide (MHW/HL 77.5%); snout short (SL/SVL 9.6%), round in dorsal profile, oval in lateral profile, extending beyond lower jaw; nostril on anterolateral margin of snout, closer to the snout tip than to the eye; labial fold absent; tongue oval, attached to anterior floor of mouth, free laterally and posteriorly; vomerine teeth series in an inverted V-shape, converging anteriorly and reaching choanae; glandular ridge on midline of crown distinct; internarial distance wider than interorbital distance (IE/IN 74.1%); head with thick dorsolateral bony ridges, prominent, slightly protruding, extending from lore to anterior end of parotoid; parotoids enlarged, crescent-shaped, projecting posteriorly with posterior margins curved medially; glandular vertebral ridge wide, well raised, slightly segmented tuberculate, extending from occiput to base of dorsal margin of tail; rib nodules large, forming knob-like warts, distinctly isolated from each other, 16 on each side of body from axilla to base of tail; gular fold present.

Limbs comparatively long, slender; length of forelimbs shorter than hindlimbs; relative length of forelimb FORE/SVL ratio 38.8%, relative length of hindlimb HIND/SVL ratio 43.0%; tips of forelimb and hindlimb overlapping when adpressed along the body; tips of fingers reaching to snout tip when foreleg is laid forward; fingers and toes well developed, free of webbing; fingers four, comparative finger lengths 1FL<4FL<2FL<3FL; toes five, comparative toe lengths 1TL<5TL<2TL<4TL<3TL.

Tail longer than the snout-vent length (TL/SVL 111.8%), compressed laterally, the base relatively broad, tapering posteriorly, tail tip pointed; tail base higher than width; fin fold narrow; tail with distinct lateral grooves; ventral side smooth.

Dorsal skin very rough, with small granules present on dorsal surfaces of head and back; flanks with dense granules; throat and dorsal surfaces of limbs with numerous tiny flat tubercles; chest and belly with tubercles shaped like transverse wrinkles; plantar surface of hands and feet with tiny grooves, forming reticulated pattern; lateral side of tail with granules; ventral side of tail smooth; cloacal region slightly swollen, vent as a longitudinal slit, edged with numerous small transverse folds.

##### Coloration in life.

In life, ground color of dorsal and ventral surfaces dark brown; dorsolateral bony ridges, parotoids, rib nodules, vertebral ridge, the peripheral area of the cloaca, tips of fingers, toes and lateral side of tail dull orange; ventral edge of the tail orange

##### Coloration in preservative.

In preservative, the specimens are blackish brown. The dull orange coloration in life has faded to beige.

##### Secondary sexual characteristics.

Males are likely smaller than females. However, this observation is based on a limited sample size and requires confirmation through additional female specimens. The female cloacal slit is short and its inner cloacal walls lack papillae. The male exhibits a long cloacal slit, with papillae present on the inner cloacal walls.

##### Distribution.

The new species is currently known only from the evergreen forest on Phu Xai Lai Leng Mountain in Nghe An Province, north-central Vietnam (Fig. 1).

##### Ecological notes.

**Figure 9.**
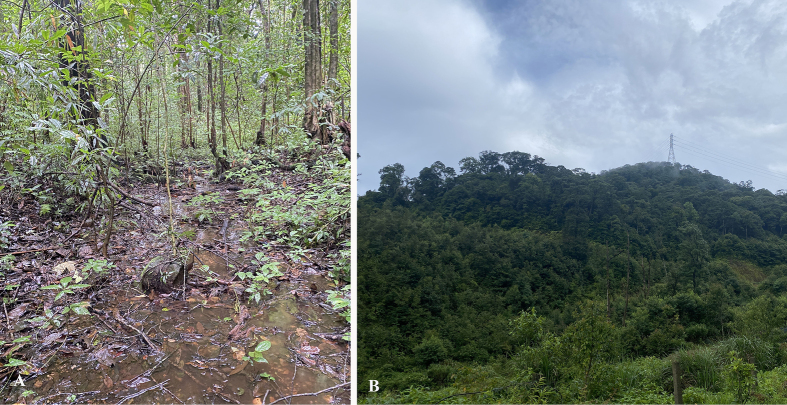
Natural habitat of *Tylototriton vietnamirabilis* sp. nov. in Phu Xai Lai Leng Mountain, Nghe An Province, Vietnam. **A**. Microhabitat; **B**. Evergreen forest. Photos: TPQ.

Type specimens were collected at night between 19:00 and 21:00 h, in a small pond (Fig. 9A). The surrounding habitat was evergreen broadleaf forest composed of large, medium, and small hardwoods mixed with shrubs (Fig. 9B). Air temperatures and relative humidity at the sites ranged from 19.1–23.5 °C and 90–98%, respectively.

##### Morphological measurements.

Morphometric measurements of the type series of *Tylototriton vietnamirabilis* sp. nov. are given in Table 2.

##### Morphological comparisons.

We compared the new species with other members of the subgenus *Tylototriton* based on data obtained from the literature (Suppl. material 2).

Morphologically, *Tylototriton vietnamirabilis* sp. nov. resembles *T. anguliceps*, a recently described species from northwestern Vietnam and Thailand. However, the new species is distinguished from the latter by having dorsolateral bony ridges on head thick (vs narrow), internarial distance wider than interorbital distance (vs narrower), parotoids, rib nodules as well as vertebral ridge dull orange (vs bright orange), dorsal surface of head dark brown (vs orange), and chin and throat dark brown (vs orange with dark markings) (Fig. 8);

from *T. himalayanus* Khatiwada, Wang, Ghimire, Vasudevan, Paudel & Jiang, 2015 by having rib nodules large (vs small), the fin fold on tail narrow (vs well developed), and parotoids, rib nodules, vertebral ridge dull orange (vs dark brown);

from *T. kachinorum* Zaw, Lay, Pawangkhanant, Gorin & Poyarkov, 2019 by having a smaller size in males (TOL 125.5–141.5 mm vs 144.5–170.3 mm), more rib nodules (15–16 vs 13–14), the glandular ridge on crown distinct (vs indistinct), the presence of lateral grooves on tail (vs absent), and tail with narrow fin fold (vs well developed);

from *T. kweichowensis* by having a smaller size in males (TOL 125.5–141.5 mm vs 155.0–195.0 mm), tail longer than the snout-vent length in males (vs shorter), the presence of gular fold (vs absent), rib nodules large and knob-like (vs small and not segmented), and tail with narrow fin fold (vs well developed);

from *T. panwaensis* by having vomerine teeth in contact with choanae (vs not in contact), the presence of gular fold (vs absent), the vertebral ridge segmented (vs not segmented), tail longer than the snout-vent length in males (vs shorter), and a greater ratio of CIL/SVL in males (0.14 vs 0.07);

from *T. phukhaensis* by having rib nodules large (vs small), vertebral ridge wide (vs narrow), the presence of lateral grooves on tail (vs absent), and tail with a narrow fin fold (vs well developed);

from *T. podichthys* by having glandular ridge on crown distinct (vs indistinct), the presence of lateral grooves on tail (vs absent), the vertebral ridge segmented (vs not segmented), and parotoids, rib nodules as well as vertebral ridge dull orange (vs bright orange), chin and throat dark brown (vs orange with dark markings);

from *T. pulcherrimus* by the presence of lateral grooves on tail (vs absent), and tail with narrow the fin fold (vs well developed), and parotoids, rib nodules as well as vertebral ridge dull orange (vs bright orange-yellow);

from *T. shanjing* by having tail longer than the snout-vent length in males (vs shorter), parotoids, rib nodules as well as vertebral ridge dull orange (vs orange-yellow), tail with narrow fin fold (vs well developed), dorsal surface of head dark brown (vs orange), and chin and throat dark brown (vs orange with dark markings);

from *T. shanorum* by having a smaller size in males (TOL 125.5–141.5 mm vs 153.0 mm), more rib nodules (15–16 vs 14), the glandular ridge on crown distinct (vs indistinct), rib nodules large (vs small), the presence of lateral grooves on tail (vs absent), and tail with narrow fin fold (vs well developed);

from *T. soimalai* by the presence of gular fold (vs absent), the vertebral ridge segmented (vs not segmented), the presence of lateral grooves on tail (vs absent), tail with narrow fin fold (vs well developed), and parotoids, rib nodules as well as vertebral ridge dull orange (vs orange);

from *T. umphangensis* by having a smaller size in males (TOL 125.5–141.5 mm vs 150.5–152.7 mm), the glandular ridge on crown distinct (vs indistinct), rib nodules large (vs small), the vertebral ridge wide (vs narrow), the presence of lateral grooves on tail (vs absent), and tail with narrow fin fold (vs well developed);

from *T. uyenoi* by having a smaller size in males (TOL 125.5–141.5 mm vs 150.0 mm), rib nodules large (vs small), the presence of lateral grooves on tail (vs absent), tail with narrow fin fold (vs well developed), and parotoids, rib nodules as well as vertebral ridge dull orange (vs orange to reddish brown);

from *T. verrucosus* by the presence of gular fold (vs absent), rib nodules large (vs small), the vertebral ridge wide and segmented (vs narrow and not segmented), the presence of lateral grooves on tail (vs absent), and parotoids, rib nodules as well as vertebral ridge dull orange (vs reddish brown); and

from *T. yangi* by having tail longer than the snout-vent length in males (vs shorter), the presence of gular fold (vs absent), and parotoids, rib nodules as well as vertebral ridge dull orange (vs brownish yellow on parotoids and yellow on rib nodules, vertebral ridge).

## Discussion

*Tylototriton vietnamirabilis* exhibits the closest geographic proximity to *T. podichthys* from Laos, with a distributional distance of approximately 147 km. However, the genetic divergence between the two species is 6.45–6.47% (Phimmachak et al. 2015). In contrast, *T. vietnamirabilis* shows the lowest genetic divergence with *T. panwaensis* (5.33–5.35%), a species described from Kachin State, Myanmar (Grismer et al. 2019), at a geographic distance of 930 km. Furthermore, *Tylototriton vietnamirabilis* differs genetically from *T. anguliceps* by 5.35–5.37%, with a geographic distance of 386 km from the type locality in Muong Nhe Nature Reserve, Dien Bien Province (Le et al. 2015). The type locality of *Tylototriton vietnamirabilis* is located in the border area between Vietnam and Laos, and it is expected that the new species can be found in montane forests in Xieng Khouang and Huaphan provinces of Laos as well. A similar adult specimen from the Huaphan Province showing the same morphological characteristics as the new taxon was photographed in Hernandez (2016: 155).

*Tylototriton vietnamirabilis* represents the second species of the genus recorded from Nghe An Province, following *T. thaiorum* (Poyarkov et al. 2021), a species previously identified as *T. notialis*, and is the second member of the subgenus *Tylototriton* documented in Vietnam, after *T. anguliceps* (Le et al. 2015). The species description brings the total number of known species in the genus *Tylototriton* from Vietnam to 10. This finding further highlights the considerable diversity potential of crocodile newts in Vietnam, underscoring the need for continued research, particularly in the montane regions of Central Vietnam. *Tylototriton vietnamirabilis* is currently known only from Phu Xai Lai Leng Mountain, implying a restricted distribution range composed of a single small isolated mountain population, which is distinct evidence for a high threat potential. In addition to its special zoogeographic situation and rarity, the particular colorful appearance of the new crocodile newt species is very likely to draw the interest of illegal collectors. Therefore, this species should be provisionally considered to be listed as Endangered (EN) under IUCN Red List criteria B1ab(i,iii), as it is known only from Phu Xai Lai Leng Mountain in Nghe An Province; the estimated extent of occurrence (EOO) is less than 200 km^2^; and the species’ habitat is currently being degraded due to human impacts, for example forest product exploitation, tourism development, and agricultural cultivation. All species of the genus *Tylototriton* are listed in the appendix II of the Convention on International Trade in Endangered Species of Wild Fauna and Flora (CITES 2026) and also in the Circular 85/2025/TT-BNNMT of the Ministry of Agriculture and Environment of Vietnam (2025); and therefore, the new species is automatically protected under these regulations.

## Supplementary Material

XML Treatment for Tylototriton vietnamirabilis

## Additional information

### Artificial Intelligence (AI) use

The authors accept full responsibility for the content of the manuscript, including the disclosure of any use of AI.

No AI tools were used in the preparation of this manuscript.

### Author ORCIDs

An Vinh Ong https://orcid.org/0000-0003-3683-3832

Tien Quang Phan https://orcid.org/0000-0002-2738-5364

Chung Van Hoang https://orcid.org/0000-0002-0709-974X

Mai Hong Thi Nguyen https://orcid.org/0009-0001-8322-010X

Tao Thien Nguyen https://orcid.org/0000-0002-5640-4536

Thomas Ziegler https://orcid.org/0000-0002-4797-609X

Truong Quang Nguyen https://orcid.org/0000-0002-6601-0880

Cuong The Pham https://orcid.org/0000-0001-5158-4526

### Data availability

All of the data that support the findings of this study are available in the main text or Supplementary Information.

## Supplementary materials

Supplementary material 1 Samples of the subgenus *Tylototriton* Data type docx Explanation note Samples of the subgenus *Tylototriton* species and other species used for DNA analyses in this study. https://binary.pensoft.net/file/1587530This dataset is made available under the Open Database License (http://opendatacommons.org/licenses/odbl/1.0/). The Open Database License (ODbL) is a license agreement intended to allow users to freely share, modify, and use this Dataset while maintaining this same freedom for others, provided that the original source and author(s) are credited. An Vinh Ong, Tien Quang Phan, Chung Van Hoang, Mai Hong Thi Nguyen, Tao Thien Nguyen, Thomas Ziegler, Truong Quang Nguyen, Cuong The Pham

Supplementary material 2 Morphological comparisons Data type xlsx Explanation note Morphological comparisons between *Tylototriton vietnamirabilis* sp. nov. with other members of the subgenus *Tylototriton* (morphological data obtained from Nussbaum et al. (1995), Nishikawa et al. (2013, 2014, 2015), Khatiwada et al. (2015), Le et al. (2015), Phimmachak et al. (2015), Fei and Ye (2016), Hernandez (2016), Grismer et al. (2018, 2019), Zaw et al. (2019), and Pomchote et al. (2020, 2021, 2024). For abbreviations, see Material and methods, / = characteristics unobtainable from literature. https://binary.pensoft.net/file/1587531This dataset is made available under the Open Database License (http://opendatacommons.org/licenses/odbl/1.0/). The Open Database License (ODbL) is a license agreement intended to allow users to freely share, modify, and use this Dataset while maintaining this same freedom for others, provided that the original source and author(s) are credited. An Vinh Ong, Tien Quang Phan, Chung Van Hoang, Mai Hong Thi Nguyen, Tao Thien Nguyen, Thomas Ziegler, Truong Quang Nguyen, Cuong The Pham
