# Supplementary material for: A new species of Tylototriton (Amphibia, Salamandridae) from Phu Xai Lai Leng Mountain, Nghe An Province, Vietnam
Source: Zookeys. 2026 Apr 8;1276:285–305. doi: 10.3897/zookeys.1276.173848 (PMC13084263; doi:10.3897/zookeys.1276.173848)
Supplement: Supplementary material 1 — Samples of the subgenus Tylototriton [file zookeys-1276-285_article-173848__-s001.docx]

**Supplementary material 1.** Samples of the *Tylototriton* species and other species used for DNA analyses in this study.

| **Sample no.** | **Species** | **Voucher no.** | **Locality** | **GenBank acc. no.** | **Source** |
| --- | --- | --- | --- | --- | --- |
| 1 | *Tylototriton vietnamirabilis* sp. nov. | IB A.6427 | Phu Xai Lai Leng Mountain, Nghe An, Vietnam | **PX668414** | This study |
| 2 | *Tylototriton vietnamirabilis* sp. nov. | IB A.6428 | Phu Xai Lai Leng Mountain, Nghe An, Vietnam | **PX668413** | This study |
| 3 | *Tylototriton vietnamirabilis* sp. nov. | IBA.6429 | Phu Xai Lai Leng Mountain, Nghe An, Vietnam | **PX668412** | This study |
| 4 | *Tylototriton vietnamirabilis* sp. nov. | IB A.6425 | Phu Xai Lai Leng Mountain, Nghe An, Vietnam | **PX668415** | This study |
| 5 | *Tylototriton vietnamirabilis* sp. nov. | IB A.6426 | Phu Xai Lai Leng Mountain, Nghe An, Vietnam | **PX668411** | This study |
| 6 | *Tylototriton ngoclinhensis* | IEBR A.5132 | Ngoc Linh Mountain, Kon Tum, Vietnam | **PX668417** | This study |
| 7 | *Tylototriton ngoclinhensis* | IEBR A.5134 | Ngoc Linh Mountain, Kon Tum, Vietnam | **PX668416** | This study |
| 8 | *Tylototriton anguliceps* | VNMM A.2014.3 | Muong Nhe, Dien Bien, Vietnam | LC017832 | Le et al. (2015) |
| 9 | *Tylototriton himalayanus* | MVZ no number | Nepal | DQ517854 | Weisrock et al. (2006) |
| 10 | *Tylototriton kachinorum* | ZMMU A5953 | Indawgyi, Kachin, Myanmar | MK097273 | Zaw et al. (2019) |
| 11 | *Tylototriton kweichowensis* | MVZ 230371 | Daguan, Yunnan, China | DQ517851 | Weisrock et al. (2006) |
| 12 | *Tylototriton panwaensis* | CAS 245418 | Panwa, Myitkyina, Myanmar | KT340279 | Grismer et al. (2018) |
| 13 | *Tylototriton panhai* | No voucher | Phu Luang Wildlife Sanctuary, Loei, Thailand | AB830736 | Nishikawa et al. (2013a) |
| 14 | *Tylototriton podichthys* | KUHE 34399 | Xam Neua, Houa Phan, Laos | AB830732 | Nishikawa et al. (2013a) |
| 15 | *Tylototriton pulcherrimus* | KUHE 46406 | Yunnan, China | AB830738 | Nishikawa et al. (2013a) |
| 16 | *Tylototriton phukhaensis* | CUMZ-A-7719 | Doi Phu Kha National Park, Nan, Thailand | MN912575 | Pomchote et al. (2020b) |
| 17 | *Tylototriton shanjing* | NMNS 3682 | Jingdong, Yunnan, China | AB830721 | Nishikawa et al. (2013a) |
| 18 | *Tylototriton shanorum* | CAS 230940 | Taunggyi, Shan, Myanmar | AB922823 | Nishikawa et al. (2014) |
| 19 | *Tylototriton soimalai* | CUMZ-A-8253 | Mae Tuen Wildlife Sanctuary, Tak, Thailand | PQ218721 | Pomchote et al. 2024 |
| 20 | *Tylototriton soimalai* | CUMZ-A-8254 | Mae Tuen Wildlife Sanctuary, Tak, Thailand | PQ218722 | Pomchote et al. 2024 |
| 21 | *Tylototriton soimalai* | CUMZ-A-8256 | Mae Tuen Wildlife Sanctuary, Tak, Thailand | PQ218723 | Pomchote et al. 2024 |
| 22 | *Tylototriton taliangensis* | KUHE 43361 | Unknown, China | AB769543 | Nishikawa et al. (2013b) |
| 23 | *Tylototriton umphangensis* | CUMZ-A-8243 | Umphang Wildlife Sanctuary, Tak, Thailand | OK092618 | Pomchote et al. (2021b) |
| 24 | *Tylototriton uyenoi* | KUHE 19147 | Doi Suthep, Chiang Mai, Thailand | AB830733 | Nishikawa et al. (2013a) |
| 25 | *Tylototriton verrucosus* | KIZ 201306055 | Husa, Yunnan, China | AB922818 | Nishikawa et al. (2014) |
| 26 | *Tylototriton yangi* | KUHE 42282 | Yunnan, China | AB769546 | Nishikawa et al. (2013b) |
|  | **Outgroup** |  |  |  |  |
| 27 | *Echinotriton andersoni* | KUHE no number | Nago, Okinawa, Japan | AB769545 | Nishikawa et al. (2013b) |
